# Nuclear Factor Erythroid 2-Related Factor 2 and SARS-CoV-2 Infection Risk in COVID-19-Vaccinated Hospital Nurses

**DOI:** 10.3390/vaccines13070739

**Published:** 2025-07-09

**Authors:** Stefano Rizza, Luca Coppeta, Gianluigi Ferrazza, Alessandro Nucera, Maria Postorino, Andrea Quatrana, Cristiana Ferrari, Rossella Menghini, Susanna Longo, Andrea Magrini, Massimo Federici

**Affiliations:** 1 Department of Systems Medicine, University of Rome Tor Vergata, 00133 Rome, Italy; stefano.rizza@uniroma2.it (S.R.); g.ferrazza@hotmail.com (G.F.); alessandro.nucera@ptvonline.it (A.N.); mariapostorino@hotmail.it (M.P.); a.quatrana@outlook.com (A.Q.); menghini@uniroma2.it (R.M.); susanna.longo@uniroma2.it (S.L.); federicm@uniroma2.it (M.F.); 2 Department of Occupational Medicine, University of Rome Tor Vergata, 00133 Rome, Italy; luca.coppeta@uniroma2.it (L.C.); andrea.magrini@uniroma2.it (A.M.)

**Keywords:** COVID-19, SARS-CoV-2, healthcare workers, vaccine, BNT162b2 mRNA vaccine, anti-S-RBD IgG antibody

## Abstract

**Background/Objectives**: The COVID-19 pandemic has caused sickness and death among many health care workers. However, the apparent resistance of health care workers to SARS-CoV-2 infection despite their high-risk work environment remains unclear. To investigate if inflammation and circadian disruption contribute to resistance or diminished susceptibility to the SARS-CoV-2 virus, we retrospectively evaluated a cohort of volunteer hospital nurses (VHNs). **Methods**: A total of 246 apparently healthy VHNs (mean age 37.4 ± 5.9 years) who had received the BNT162b2 mRNA vaccine were asked to report their sleep quality, according to the Pittsburgh Sleep Quality Index, and number of SARS-CoV-2 infections during the observational study period (from the end of December 2020 to April 2025). The expression of inflammation-associated mediators and circadian transcription factors in peripheral blood mononuclear cells, as well as sleep quality, were examined. **Results**: Our findings revealed no anthropometric, biochemical, or inflammation-associated parameters but demonstrated significantly greater levels of NFE2L2, also known as nuclear factor erythroid-derived 2-like 2 (NFR2), gene expression in peripheral blood mononuclear cells among VHNs who had never been infected with SARS-CoV-2 (*n* = 97) than in VHNs with only one (*n* = 119) or with two or more (*n* = 35) prior SARS-CoV-2 infections (*p* < 0.01). This result was confirmed through one-to-one propensity score matching (*p* < 0.01). Moreover, NRF2 gene expression was not associated with the number of COVID-19 vaccinations (*p* = 0.598). Finally, NRF2 gene expression was higher among participants who reported better sleep quality (*p* < 0.01). **Conclusions**: Our findings suggest possible interactions among NRF2 gene expression, protection against SARS-CoV-2 infection, and the modulation of COVID-19 vaccination efficacy.

## 1. Introduction

COVID-19 may be considered an occupational disease, given that it has caused sickness and death among health care workers [1]. Data from the World Health Organization (WHO) revealed a very high prevalence of COVID-19 cases among health workers. Notably, although health care workers represent less than 3% of the population in most countries [2], 14% of COVID-19 cases have occurred among health care providers. Health care workers’ work activities persisted throughout the COVID-19 pandemic because their work was considered “essential.” Consequently, they faced daily workplace exposure and experienced a greater risk of COVID-19 and death than remote workers. However, the extent of work-related transmission has remained unclear because efforts to collect and analyze occupational data on COVID-19 cases have been limited [3]. On 1 April 2021, the Italian Government issued Decree Law no. 44, which established compulsory COVID-19 vaccination for health care workers. Accordingly, the local vaccination campaign for health care workers at Polyclinic Tor Vergata, Rome, using the Pfizer-BioNTech vaccine BNT162b2, began on 28 December 2020. COVID-19 vaccination has been widely reported to play a critical role in the body’s defense against SARS-CoV-2, particularly in controlling viral spread, mitigating severe outcomes, and providing long-term protection [4].

However, despite multiple vaccinations, some individuals have experienced multiple SARS-CoV-2 infections with varying degrees of intensity, whereas a small fraction of individuals have appeared to remain resistant to SARS-CoV-2 infection, as confirmed by repeated negative tests. Among several possible explanations, such as limited immune response to vaccination and vulnerable metabolic characteristics [5,6,7], the subtle but high levels of chronic inflammation and the circadian rhythm disruption that characterize workers in various health care sectors might potentially explain this inconsistent spread of COVID-19. Consequently, the main question guiding the study is to understand why certain individuals have remained uninfected by SARS-CoV-2. For this purpose, we have investigated the expression of clock genes (BMAL1 and REV-ERBα) because the alteration of their levels may reflect the presence of a circadian disruption that is potentially involved in the immune response to SARS-CoV-2, as well as inflammatory modulators such as IL-1β, IF-γ, and NFE2L2, also known as nuclear factor erythroid-derived 2-like 2 (NFR2). The latter, in particular, might also interact with the COVID-19 mRNA vaccine. During SARS-CoV-2 infection, NFR2 is highly dysregulated, thereby leading to the dysfunctional expression of ACE2 with increased viral entry [8]. NRF2 modulates type 1 interferon (IFN) production by impairing stimulator of interferon gene (STING) expression [9], which in turn may improve immune responses against SARS-CoV-2. Similarly, the COVID-19 mRNA vaccine has been reported to modulate both STING [10] and IFN [11]. Therefore, the elevated expression of NFR2 and the COVID-19 vaccine might also be involved in mitigating the progression of cytokine storm in COVID-19. This point is particularly intriguing considering that the COVID-19 vaccine has remained highly effective in reducing the severity of disease, including hospitalizations and mortality.

## 2. Materials and Methods

This is a retrospective study that recruited a group of volunteer hospital nurses (VHNs) at the Policlinico Tor Vergata University between 2012 and 2015, for a clinical trial, which has been extensively described elsewhere [12]. The Tor Vergata University Ethics Board approved the study protocol (decision n. EU-FP3:125/21 on 14 December 2021). Written consent was obtained from each patient after a full explanation of the purpose and nature of all study procedures used.

### 2.1. Study Participants

The trial included a population of VHNs who had worked at the Policlinico Tor Vergata University since 2012 or earlier, for a minimum of 2 years, and had performed hospital tasks of any type. We applied the exclusion criteria using clinical evaluation, medical records, and laboratory data. These included the presence of diabetes, liver disease, renal insufficiency, heart failure, coagulopathy, or any other severe systemic disease. Participants were also excluded if they had a history of any form of cancer; had positive blood tests for HIV, hepatitis B, or hepatitis C; or had taken melatonin supplements within 4 weeks before commencing the study.

In participants (*n* = 278), we recorded the body mass index (BMI, calculated as the weight in kilograms divided by the square of the height in meters), blood pressure, and smoking status.

### 2.2. Sleep Quality Analysis

We assessed the sleep quality reported by participants using the Pittsburgh Sleep Quality Index (PSQI), a validated scale that can identify elements of sleep [13]. This questionnaire identifies seven “components” of sleep routinely assessed clinically: sleep latency, sleep duration, habitual sleep efficiency, sleep disturbance, the use of sleep medication, daytime dysfunction, and subjective sleep quality. The sum of the scores of the aforementioned seven components yields a global PSQI score that ranges from 0 to 21 points; a score of 5 or greater is associated with poor sleep quality and was therefore dichotomized as such in our analysis. The PSQI scores have been shown to have good test–retest reliability, with a correlation coefficient of 0.85 for the global score and correlation coefficients ranging from 0.65 (medication use) to 0.84 (sleep latency) for the component scores.

### 2.3. Clock Gene Analysis

During the visit performed between 2012 and 2015, after an overnight fast, approximately 20 mL of whole blood was collected from all study participants. Approximately 8 mL was used for the extraction of PMBC RNA and a quantitative real-time PCR analysis, as previously described [12]. Briefly, single-strand cDNA was synthesized according to the Applied Biosystems (Foster City, CA, USA) standard protocol from 2 μg total RNA samples with a High-Capacity cDNA Archive Kit. Subsequently, 50 ng cDNA was amplified via real-time PCR using TaqMan Master mix (Applied Biosystems; Thermo Fisher Scientific, Foster City, CA, USA) with an ABI PRISM 7500 system. The thermocycling steps were as follows: 95 °C for 10 min, followed by 39 cycles of 95 °C for 30 s, 60 °C for 1 min, and 65 °C for 30 s. The relative mRNA expression was calculated using the 2^−ΔΔCt^ method. The results were normalized to 18S rRNA as an endogenous control. The primer identification numbers (Applied Biosystems) were as follows: BMAL1: Hs00154147¬_m1; REV-ERBα: Hs00253876_m1; IL-1β: Hs00174097_m1; IF-γ: Hs00989291_m1; and NFR-2: Hs00975961_g1.

### 2.4. COVID-19 Vaccination

Beginning on 28 December 2020, after providing informed consent, most study participants received the scheduled vaccination series for COVID-19. The Pfizer-BioNTech vaccine BNT162b2 was administered intramuscularly in a series of two doses (0.3 mL each) 3 weeks apart, with indications for booster administration to four or five doses in total.

A few individuals (approximately 3.5% of Policlinico Tor Vergata health care workers) did not receive the COVID-19 vaccination because of personal choice or vaccine hesitancy.

### 2.5. SARS-CoV-2 Infection

All participants routinely underwent an annual hospital visit at the Occupational Medicine Service of the University of Tor Vergata, where operators collected a medical history, performed a full clinical examination, and collected information about the number and clinical severity of SARS-CoV-2 infections acquired after COVID-19 vaccination. Data were reported for each positive nasopharyngeal swab test for SARS-CoV-2. To confirm infections, on 2 January 2025 and 2 March 2025, the study population was asked to participate in serum collection for anti-S-RBD IgG antibody testing with a Roche “Elecsys^®^” kit (Roche, Rotkreuz, Switzerland). Positive results were identified according to levels exceeding 0.8 U/mL, according to the manufacturer’s instructions. Therefore, the total observation study period began by the end of 2020, when the first Pfizer-BioNTech BNT162b2 mRNA vaccine was administered. The observation period ended in April 2025.

### 2.6. Statistical Analysis

Continuous data were tested for skewness via the visual inspection of QeQ plots, stem and leaf plots, or box plots, as well as with the Shapiro–Wilk test for normal distributions. Quantitative data are reported as mean ± standard deviation, whereas categorical variables are reported as numbers of participants. We compared the mean values or frequencies of study variables among groups using analysis of variance, the Kruskal–Wallis test, the impaired *t*-test, or the Mann–Whitney U test as appropriate. Post hoc comparisons were performed using Bonferroni’s test. Subsequently, we used one-to-one propensity score matching of individuals between groups with or without prior SARS-CoV-2 infection. The propensity score was estimated with logistic regression using age, sex, and smoking status as predictors, and a caliper of 0.015 was applied to ensure that participants with similar propensity scores were appropriately matched. For the logistic regression used in propensity score, we set the maximum number of predictors to three, according to a common heuristic rule of having approximately one predictor per 70–80 individuals. A higher ratio may cause imprecise and ungeneralizable estimates.

All tests were two-sided, and the threshold for statistical significance was set at *p* ≤ 0.05. All analyses were performed in *SPSS* version 19.0 for Windows.

## 3. Results

Among the 278 participants, we excluded 4 VHNs because they did not receive a COVID-19 vaccination, 18 individuals for whom the exact number of positive nasopharyngeal swabs for SARS-CoV-2 was not reported, and 10 VHNs who refused blood withdrawal for SARS-CoV-2 IgG antibody testing. Overall, our results revealed a higher incidence of SARS-CoV-2 infection among young hospital nurses (about 60% of study participants). This could be related to less experience in protective device use, as well as greater social activity outside the hospital.

The mean age of the included study participants (*n* = 246) was 37.4 ± 5.9 years. Most participants were women (*n* = 184) and nonsmokers (*n* = 161). According to the Occupational Medicine Service of the University of Tor Vergata reports, we divided participants into three groups according to the presence and number of previous SARS-CoV-2 infections: never infected VHNs (*n* = 97), VHNs with one prior SARS-CoV-2 infection (*n* = 119), and VHNs with two or more SARS-CoV-2 infections (*n* = 35) (Table 1).

**Figure 1:**
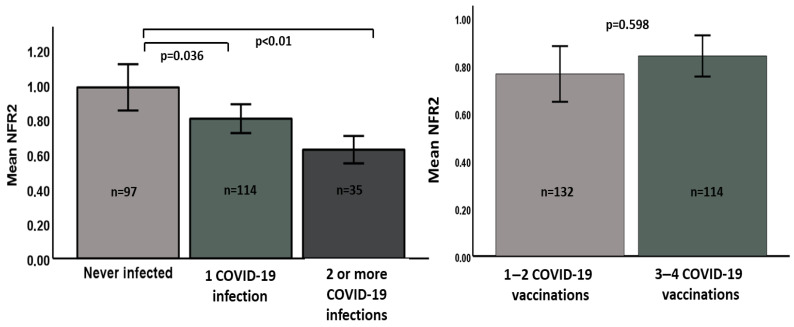
Left panel: NFR-2 mRNA levels in different groups based on the number of SARS-CoV-2 infections. *p* value between bars refers to post hoc comparisons using Bonferroni’s test. Right panel: NFR-2 mRNA levels in subjects divided according to the number of COVID-19 vaccinations.

The three groups did not differ in terms of age, smoking status, glucose and lipid profiles, and kidney and liver function. Notably, although it was not statistically significant, participants with two or more SARS-CoV-2 infections showed higher BMI and systolic blood pressure, but not diastolic blood pressure, than the other two groups of VHNs. Furthermore, the mRNA levels of CRP, IFN-ɣ, IL-1β, and clock genes did not differ among groups. Interestingly, the NRF2 mRNA levels were statistically higher in the never-infected group than in the other groups (Table 1, Figure 1, *p* < 0.01), thus suggesting an association between lower levels of this factor and a greater likelihood of infection recurrence. As shown in Figure 1, panel left, pair-wise comparisons based on post-hoc Bonferroni’s tests confirmed that never-infected participants had significantly higher NFR-2 mRNA levels than individuals with one SARS-CoV-2 infection (*p* = 0.036) and participants with two or more SARS-CoV-2 infections (*p* < 0.001). The NFR2 level showed a much weaker and not significant association with the number of COVID-19 vaccinations (Figure 1, right panel).

Notably, the four VHNs without COVID-19 vaccinations had similar NFR-2 mRNA levels to the rest of the study participants (0.9 ± 0.6 AU).

**Figure 2:**
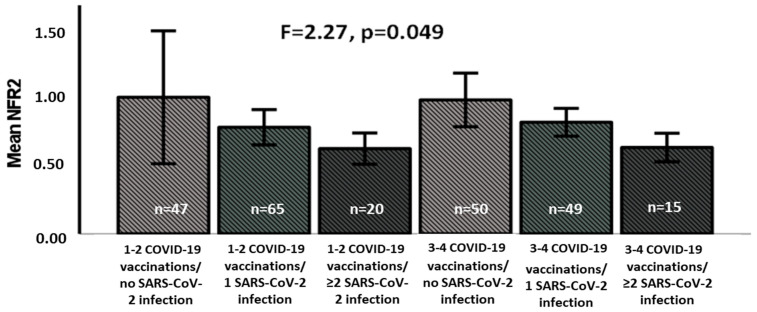
NFR-2 mRNA levels in different groups based on SARS-CoV-2 infections and COVID-19 vaccinations.

To better interpret the differing levels of NFR-2 among study participants, we divided the study cohort into six groups according to the number of SARS-CoV-2 infections and COVID-19 vaccinations: VHNs with one or two COVID-19 vaccinations who were never infected (*n* = 47); VHNs with one or two COVID-19 vaccinations and one infection (*n* = 65); VHNs with one or two COVID-19 vaccinations and two or more SARS-CoV-2 infections (*n* = 20); VHNs with three or four COVID-19 vaccinations who were never infected (*n* = 50); VHNs with three or four COVID-19 vaccinations and one infection (*n* = 49); and VHNs with three or four COVID-19 vaccinations and two or more infections (*n* = 15). We observed a significantly different distribution of NFR2 expression levels among the six groups of participants. However, as the bars in Figure 2 show, the number of COVID-19 infections was confirmed to be related to NFR2 levels, regardless of the number of COVID-19 vaccinations.

To overcome potential bias due to the numeric imbalance between participants with COVID-19 who were never infected (*n* = 97) and those with one or more SARS-CoV-2 infections (*n* = 149), we applied propensity score matching using age, sex, and smoking status as covariates. Furthermore, 56 participants were excluded: 54 in the no-infection group and 2 in the SARS-CoV-2 infection group. Consequently, we obtained two well-balanced groups of 95 participants each (Table 2). The distributions of propensity scores between COVID-negative and COVID-positive participants were perfectly aligned (*p* = 0.901). The two groups did not show any significant differences in study variables with respect to age, smoking status, blood pressure, glucose and lipid profiles, inflammation, kidney and liver function, and clock gene mRNA levels. However, BMI and NFR-2 mRNA expressions were imbalanced between groups. In particular, never-infected participants had lower BMI values than participants with prior SARS-CoV-2 infection (24.2 ± 3.5 vs. 26.3 ± 4.2, *p* < 0.01). Furthermore, the difference between the NFR-2 mRNA levels of never-infected participants and those with a history of SARS-CoV-2 infection remained statistically significant (0.99 ± 0.59 vs. 0.75 ± 0.41, *p* < 0.01). These findings further supported the finding that elevated NFR-2 mRNA expression in VHNs protected against SARS-CoV-2 infection. Finally, quantitative evaluation revealed lower NFR-2 gene expression in PBMCs from participants with poor sleep quality compared to those with good sleep quality (0.77 ± 0.56 vs. 0.98 ± 0.61, *p* < 0.01).

## 4. Discussion

Our study indicated an association between NFR-2 gene expression and SARS-CoV-2 viral infection. High expression of NFR-2 mRNA in PMBCs was associated with diminished SARS-CoV-2 infection risk among young and apparently healthy hospital nurses. NFR-2 levels were lower in participants with two or more SARS-CoV-2 re-infections than in those who had never been infected with SARS-CoV-2. To overcome possible influences of the lack of randomization, we applied propensity score matching, controlling for age, sex, and smoking status—variables that are potentially associated with SARS-CoV-2 infection. Our main findings persisted after this statistical procedure. To better interpret the association between NFR-2 expression and SARS-CoV-2 infection risk, we also considered the number of Pfizer vaccinations [14] received by the study population; greater numbers of COVID-19 vaccinations did not appear to confer greater protection against reinfection among vaccinated participants with comparable NFR-2 expression.

Our study remains an observational report; therefore, we cannot generate strong conclusions about why certain individuals remained uninfected despite continually working during the COVID-19 pandemic. However, our main results provide several novel observations. The group of hospital nurses who remained uninfected, compared with the other groups, had higher expression of NFR-2, a gene that is clearly involved in protection against viral infection, yet has not been previously described in the clinical context of SARS-CoV-2 infection. In fact, although several studies have been aimed at searching for protective mechanisms based on suppressing viral replication [15], and the activation of NFR-2 has been postulated to have a protective role against infectious diseases [16], only recently have a few non-clinical studies indicated that NFR-2 is involved in cellular defense from SARS-CoV-2 infection [16]. Viruses are well known to use cellular metabolism to replicate their genomes and produce viral proteins and several intracellular factors. These products, in addition to modifying the cellular redox state, directly or indirectly affect the progression and outcome of viral infection. In this context, NFR-2 acts as a cytoprotective enzyme with antioxidant and anti-inflammatory roles [17,18,19]. NFR-2, a transcription factor involved in regulating HMOX1, dissociates from its ligand, KEAP-1 (kelch-like ECH-associated protein), in the cytoplasm and subsequently translocates to the nucleus, where it interacts with the Small Maf Protein and activates HMOX1 gene expression. In addition, increased NRF2 activity restrains mitochondrial ROS production, conveys fatty acid oxidation, and promotes mitochondrial integrity by enhancing mitophagy [20]. Therefore, Nrf2 activators can attenuate endothelial dysfunction, renin–angiotensin system dysregulation, immune thrombosis, and coagulopathy [21]. In contrast, both the nuclear content [22] and functional activity of NRF-2 are diminished in aged animals [23], as aging is a well-known driver of reduced mitochondrial function, the dysregulation of cellular redox status, and chronic systemic inflammation [24]. Accordingly, genetic mutations in NFR-2 are associated with an inconsistent anti-inflammatory response [25]. Despite the lack of a biological explanation, we speculate that in individuals with innate anti-viral mechanisms, due partly to high expression of NFR-2, this cytoprotective enzyme with antioxidant and anti-inflammatory roles might potentially positively regulate immune responses. In fact, NFR-2, which is involved in maintaining cellular homeostasis, regulates the expression of genes involved in detoxification and cellular defense mechanisms, thereby increasing cells’ overall resilience and ability to counteract viral infections. The lack of interaction between NFR2 expression levels and COVID-19 vaccination is somewhat surprising because the COVID-19 mRNA vaccine has been reported to reduce the risk of SARS-CoV-2 infection, the severity of disease, hospitalizations, and mortality; NFR2 also has antiviral properties that act through multiple pathways [26]. A possible explanation may be based on the immune response to SARS-CoV-2 that involves both innate and adaptive immunity. It is possible that NFR2 expression levels did not significantly change virus-neutralizing antibody production after COVID-19 vaccination, thereby failing to modulate the antigen-specific responses of B and T cells.

Our data confirm a recent report [27] that identified particular innate immune responses as being responsible for resistance to COVID-19 infection among health care workers. The authors found transcriptomic signatures involving IFN-associated innate immune responses that protect against SARS-CoV-2 infection. Interestingly, NFR2 acts by modulating IFN production by affecting STING expression [9], which in turn may improve immune responses against SARS-CoV-2. However, to the best of our knowledge, strong data from clinical studies remain lacking.

Another notable finding was the correlation between NFR-2 expression and sleep quality, according to the PSQI, which is an assessment scale that provides a reliable, valid, and standardized measure of the quality of sleep. Interestingly, participants with greater NFR-2 expression had better sleep quality. This relationship might have substantial effects on preventing SARS-CoV-2 infection. The circadian rhythm disruption or sleep deprivation frequently observed among hospital workers [8] might potentially have affected NFR-2 RNA expression in PBMCs among our study participants. Interestingly, our findings are in line with those of prior reports suggesting that poorer sleep quality might lead to the downregulation of NRF2 expression in both animals [28] and humans [29]. In addition, because sleep affects the innate and adaptive immune system, good sleepers with high NFR-2 expression might achieve better vaccine responses, thereby promoting host defense against SARS-CoV-2 infection. Our results revealed that REV-ERBα and BMAL1 mRNA expression in PBMC was similar in participants with two or more SARS-CoV-2 re-infections and in those who were never infected with SARS-CoV-2. This result is surprising because clock genes are indicated as a potential marker of desynchronization of the master clock caused by strong workload during prolonged shifts and poor quality of sleep [30,31]. Therefore, whether the circadian misalignment and the disruption of rhythmic expression of clock genes (Rev-ERBα and BMAL1) in hospital workers could lead to a significant risk of SARS-CoV-2 infection remains to be determined.

### Study Limitations and Strengths

Our study has several weaknesses. First, the small number of participants might have influenced our results, and a control group has not been considered. Second, the calculation of the number of SARS-CoV-2 infections was based on self-reported events, and therefore, possible uncertainty in the data cannot be excluded. However, SARS-CoV-2 infection was confirmed through anti-S-RBD IgG antibody serum levels. Third, because of the study’s observational nature, the findings suggest only simple associations but cannot exclude the possibility that this enzyme might only be a mediator of broader, more complex mechanisms. Finally, we did not correct our findings for potentially significant confounders, such as occupational exposure intensity, night shift work, and genetic background. All of these covariates might bias the study results.

However, certain characteristics of this study are notable. First, the participants were young and apparently healthy hospital nurses with no known risk factors for SARS-CoV-2 infection that might have influenced the efficacy of COVID-19 vaccination. This aspect was particularly important in avoiding the confounding of the study results. Moreover, although the participant distribution was initially imbalanced among groups and the participants were not randomized, through propensity score matching, we created groups that were very similar in terms of several important clinical variables often associated with SARS-CoV-2 infection risk. Finally, to the best of our knowledge, this study is the first report to directly relate SARS-CoV-2 infection risk to NFR-2 levels in PBMCs, which is a heterogeneous population of blood cells that includes lymphocytes.

## 5. Conclusions

The protection of hospital care workers represents a public health priority, as the failure to protect the health and safety of frontline operators enhances the risk of collapse of the health system and may lead to the spread of the infection from health care settings to the community. In this context, the main results of our study reveal a possible new marker of SARS-CoV-2 infection risk among hospital workers. The levels of NFR-2, a cytoprotective enzyme with antioxidant and anti-inflammatory roles that potentially positively regulate immune responses, dosed in PBMCs, may indicate the innate ability of the immune system to counteract SARS-CoV-2 infection. Nevertheless, the possible interactions among NFR2 gene expression, protection against SARS-CoV-2 infection, and the modulation of the efficacy of COVID-19 vaccination in a health care setting warrant further validation in larger community populations. For this purpose, we are planning to validate these findings in independent cohorts. We will explore different strategies, such as specific diets [32] or physical activity, aimed at both improving metabolism regulation and modulating NFR2 levels.

## Figures and Tables

**Table 1:** Clinical and laboratory characteristics of patients divided according to the number of SARS-CoV-2 infections. Data are shown as numbers or means (±SD).

	Overall (*n* = 246)	Never Infected (*n* = 97)	One Infection (*n* = 114)	Two or More Infections (*n* = 35)	*p*
Age (years)	37.2 ± 6.0	37.0 ± 6.6	37.9 ± 6.1	37.4 ± 5.2	0.652
Sex (female/male)	184/62	67/30	88/26	29/6	0.198
Smoke (current-former vs. never)	85/161	33/64	40/74	12/23	0.229
BMI	24.6 ± 4.6	24.3 ± 3.9	24.4 ± 4.2	26.1 ± 5.1	0.273
Systolic Blood Pressure (mmHg)	112.5 ± 12.2	110.3 ± 12.3	112.1 ± 12.1	115.0 ± 12.5	0.314
Diastolic Blood Pressure (mmHg)	73.8 ± 8.7	72.1 ± 8.1	74.4 ± 9.7	74.3 ± 9.4	0.301
Fasting glucose (mg/dL)	88.5 ± 8.2	88.5 ± 8.2	88.9 ± 8.3	88.2 ± 8.9	0.789
HbA1c (%)	5.3 ± 0.3	5.3 ± 0.3	5.3 ± 0.3	5.3 ± 0.3	0.800
Total cholesterol (mg/dL)	188.3 ± 35.9	186.5 ± 36.0	192.2 ± 34.9	194.6 ± 36.6	0.364
HDL (mg/dL)	59.5 ± 15.8	59.5 ± 17.5	60.3 ± 13.6	58.5 ± 16.0	0.931
LDL (mg/dL)	110.4 ± 30.2	107.2 ± 30.7	112.4 ± 30.8	116.2 ± 29.0	0.314
Triglycerides (mg/dL)	98.8 ± 77.3	99.6 ± 75.6	97.1 ± 55.8	99.5 ± 87.9	0.907
Creatinin (mg/dL)	0.8 ± 0.1	0.8 ± 0.1	0.8 ± 0.1	0.8 ± 0.2	0.849
Aspartate aminotransferase (AST) (U/L)	14.4 ± 7.2	14.9 ± 7.9	14.4 ± 6.5	14.0 ± 5.5	0.724
Alanine aminotransferase (ALT) (U/L)	27.8 ± 14.2	27.0 ± 11.6	27.2 ± 14.0	28.7 ± 15.0	0.929
CRP (mg/L)	1.7 ± 2.7	1.9 ± 3.6	1.5 ± 2.5	1.8 ± 2.5	0.916
REV-ERBα mRNA (AU)	1.96 ± 1.32	2.01 ± 1.45	1.84 ± 0.57	2.00 ± 1.54	0.331
B-MAL1 mRNA (AU)	1.10 ± 0.51	1.02 ± 0.61	1.11 ± 0.52	1.18 ± 0.31	0.451
REV-ERBα/BMAL1	1.75 ± 0.72	1.99 ± 0.79	1.65 ± 0.32	1.69 ± 1.86	0.302
IFN-ɣ mRNA (AU)	0.8 ± 0.8	0.9 ± 0.8	0.8 ± 0.8	0.7 ± 0.5	0.303
IL-1β mRNA (AU)	7.3 ± 17.2	8.1 ± 20.0	7.9 ± 17.3	4.4 ± 6.7	0.544
NFR-2 mRNA (AU)	0.8 ± 0.4	1.0 ± 0.6	0.8 ± 0.4	0.6 ± 0.2	<0.01

Notes: *p*-values refer to analysis of variance or the Kruskal–Wallis test for quantitative variables, as appropriate. For categorical variables, *p*-values were generated by the chi-square test.

**Table 2:** Clinical and laboratory characteristics of patients after propensity score matching. Data are shown as numbers or means (±SD).

	No SARS-CoV-2 Infection (*n* = 95)	SARS-CoV-2 Infection (*n* = 95)	*p*
Age (years)	37.1 ± 6.7	38.4 ± 5.9	0.159
Sex (female/male)	66/29	68/27	0.437
Smoke (current-former vs. never)	32/63	32/63	1
BMI	24.2 ± 3.5	26.3 ± 4.2	<0.01
Systolic Blood Pressure (mmHg)	110.3 ± 12.3	113.8 ± 13.3	0.601
Diastolic Blood Pressure (mmHg)	72.1 ± 8.1	75.2 ± 9.7	0.022
Fasting glucose (mg/dL)	88.6 ± 8.3	88.8 ± 7.4	0.843
HbA1c (%)	5.3 ± 0.3	5.3 ± 0.3	0.164
Total cholesterol (mg/dL)	186.7 ± 36.1	198.4 ± 35.8	0.025
HDL (mg/dL)	59.5 ± 17.6	57.6 ± 14.8	0.417
LDL (mg/dL)	107.4 ± 30.8	119.2 ± 36.6	0.010
Triglycerides (mg/dL)	98.5 ± 75.7	108.5 ± 72.2	0.351
Creatinin (mg/dL)	0.79 ± 0.15	0.79 ± 0.14	0.961
Aspartate aminotransferase (AST) (U/L)	14.9 ± 7.9	14.4 ± 6.3	0.573
Alanine aminotransferase (ALT) (U/L)	26.9 ± 11.7	29.1 ± 15.9	0.270
CRP (mg/L)	1.9 ± 3.6	1.7 ± 3.4	0.553
REV-ERBα mRNA (AU)	1.92 ± 1.61	1.83 ± 1.02	0.502
B-MAL1 mRNA (AU)	1.00 ± 0.49	1.07 ± 0.55	0.602
REV-ERBα/BMAL1	1.89 ± 0.81	1.77 ± 0.47	0.338
IFN-ɣ mRNA (AU)	0.95 ± 0.90	0.86 ± 0.86	0.472
IL-1β mRNA (AU)	8.1 ± 19.9	8.7 ± 18.6	0.846
NFR-2 mRNA (AU)	0.99 ± 0.59	0.75 ± 0.41	<0.01

Notes: Propensity score matching excluded 56 participants from the original cohort of participants: 54 in the no SARS-CoV-2 infection group and 2 in the SARS-CoV-2 infection group. *p*-values refer to the unpaired *t*-test (or Mann–Whitney U test) for mean values and the chi-square test for numbers.

## Data Availability

The original contributions presented in this study are included in the article. Further inquiries can be directed to the corresponding author.

## Abbreviations

VHNs, volunteer hospital nurses; PSQI, Pittsburgh Sleep Quality Index; NFR-2, nuclear factor erythroid 2-related factor 2; PBMCs, peripheral blood mononuclear cells; BMAL1, basic helix-loop-helix ARNT-like protein 1; REV-ERBα, Nuclear Receptor Subfamily 1 Group D Member 1; IFN-ɣ, Interferon Gamma; IL-1β, Interleukin-1 Beta.
